# Microscale Temperature Shaping Using Spatial Light Modulation on Gold Nanoparticles

**DOI:** 10.1038/s41598-019-40382-3

**Published:** 2019-03-15

**Authors:** Ljiljana Durdevic, Hadrien M. L. Robert, Benoit Wattellier, Serge Monneret, Guillaume Baffou

**Affiliations:** 1 Institut Fresnel, CNRS, Aix Marseille Univ, Centrale Marseille, Marseille, France; 2 PHASICS S.A., Parc technologique de Saint Aubin, Route de l’Orme des Merisiers, 91190 Saint Aubin, France

## Abstract

Heating on the microscale using focused lasers gave rise to recent applications, e.g., in biomedicine, biology and microfluidics, especially using gold nanoparticles as efficient nanoabsorbers of light. However, such an approach naturally leads to nonuniform, Gaussian-like temperature distributions due to the diffusive nature of heat. Here, we report on an experimental means to generate arbitrary distributions of temperature profiles on the micrometric scale (e.g. uniform, linear, parabolic, etc) consisting in illuminating a uniform gold nanoparticle distribution on a planar substrate using spatially contrasted laser beams, shaped using a spatial light modulator (SLM). We explain how to compute the light pattern and the SLM interferogram to achieve the desired temperature distribution, and demonstrate the approach by carrying out temperature measurements using quantitative wavefront sensing.

## Introduction

Heating over a microscale area is becoming an important concept with the development of nano- and microtechnologies. In particular, heating gold nanoparticles by light absorption is at the basis of a more and more active field of research named thermoplasmonics^1,2^, addressing problems in biology^3–5^, biomedicine^6–8^, microscale fluid dynamics^9,10^, phase transitions (bubbles)^11–15^, thermophoresis^16,17^ or chemistry^18–20^.

The task of measuring the temperature on the microscale is now well-mastered by numerous optical microscopy techniques^1,21^, usually based on fluorescence measurements, more rarely label-free^22–25^, sometimes even in three dimensions^26^, and with a diffraction-limited spatial resolution. However, controlling the temperature *spatial distribution* is, for the time being, overlooked. Light-heating of gold nanoparticle distributions usually results in non-uniform, Gaussian-like profiles, due to the diffusive nature of heat^27^. Yet, for some applications, the temperature spatial profile can play an important role. For instance, any temperature gradient can generate important thermophoretic forces on colloids dispersed in liquids^16,17,28^, or non-uniform temperatures may be detrimental when working with biological systems, sensitive to temperature variations of typically 0.5 K^4^. For such applications, it would be important to accurately monitor the gradients or control the uniformity of the microscale temperature profile.

In a previous work, we achieved microscale temperature shaping at will by illuminating sophisticated and non-uniform nanoparticle distributions, made by e-beam lithography, using uniform laser beams^29^. Albeit effective, this approach suffers from a lack of flexibility: each lithographied area is associated with a predefined temperature profile that could neither be dynamically changed, nor moved to any other area of interest. This may be a stringent constraint for instance in applications involving living cells in culture, whose position can never be predicted in advance.

In this article, we introduce an experimental procedure to dynamically generate distributions of temperature profiles on the micrometric scale with arbitrary complexity by illuminating a uniform gold nanoparticle distribution using spatially contrasted laser beams, shaped using a spatial light modulator (SLM). We explain hereinafter how to compute the light pattern and the SLM interferogram to achieve the desired temperature distributions, explain the benefits of using gold nanoparticles and demonstrate the approach by carrying out temperature measurements using quantitative phase imaging. A final part is dedicated to an illustration of the interest of this approach for the field of thermophoresis of colloids.

## Results and Discussion

**Figure 1:**
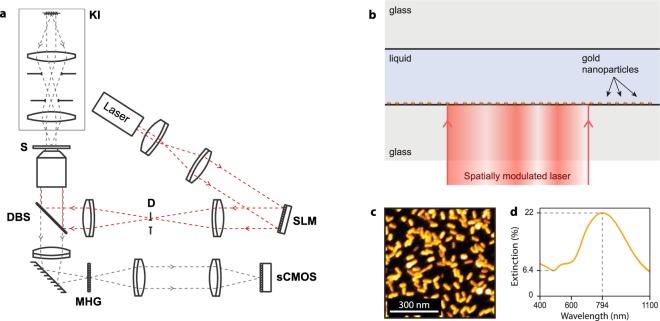
(**a**) Scheme of the microscope setup. A spatial light modulator (**SLM**) is used to create arbitrary laser beam profiles at the sample (**S**) plane, via a diaphragm (**D**, to remove the zero-order diffraction spot) and a dichroic beam splitter (**DBS**). A Köhler illumination (**KI**) is implemented on top of the sample. A QSI wavefront sensor, consisting of sCMOS camera assembled to a reimaging system and coupled with a two-dimensional grating (commonly named a Modified Hartmann Mask, **MHM**) are used to map the wavefront distorsion created by the temperature gradient resulting from the laser heating of the gold nanoparticles at the sample plane. (**b**) Schematic of the sample, which consists of a water layer sandwiched between two glass coverslips. In our study, the nanoparticles are gold nanorods. They are deposited on top of the lower coverslip, and are meant to be heated by a laser illumination through the objective lens (40×). (**c**) SEM image of the gold nanorods. (**d**) Typical extinction spectrum of the gold nanorod samples used in this study.

A single home-made microscope was used to both heat substrates of gold nanoparticles, and measure the resulting temperature distribution, on the microscale. The microscope configuration is sketched in Fig. 1a. A spatial light modulator (SLM, HSP256-1064 Boulder Nonlinear Systems) was used to shape the profile (see next subsection) of a laser beam (Ti:Sapph laser, $\lambda =800\,{\rm{nm}}$) that was sent to the sample using a dichroic beam splitter (DBS). The association of a Köhler illumination (Thorlabs LED $\lambda =625\pm 12\,{\rm{nm}}$), a modified Hartmann mask (MHM, Phasics SA), a reimaging system (Sid4-Element, Phasics) and a sCMOS camera (Zyla, Andor) were used to acquire the temperature distribution of the sample resulting from the heating of the gold nanoparticles. This label-free technique, called TIQSI for temperature imaging using quadriwave lateral shearing interferometry, is described in a previous publication^30^. This technique does not only enable the mapping of the temperature but also of the heat source density (power per unit area).

The sample is depicted in Fig. 1b. It was composed of a water layer sandwiched between two glass coverslips (1 in × 1 in). Depending on the experiments presented in this article, the water layer thickness could vary from a few microns to 1 mm. On the bottom coverslip, a uniform layer of gold nanorods ($46\pm 4\,{\rm{nm}}$ in length and $20\pm 2\,{\rm{nm}}$ in diameter) featuring a plasmonic resonance around the laser wavelength (800 nm) was deposited over the whole area of the coverslip (see ref.^4^ for details regarding the fabrication of this kind of sample).

**Figure 2:**
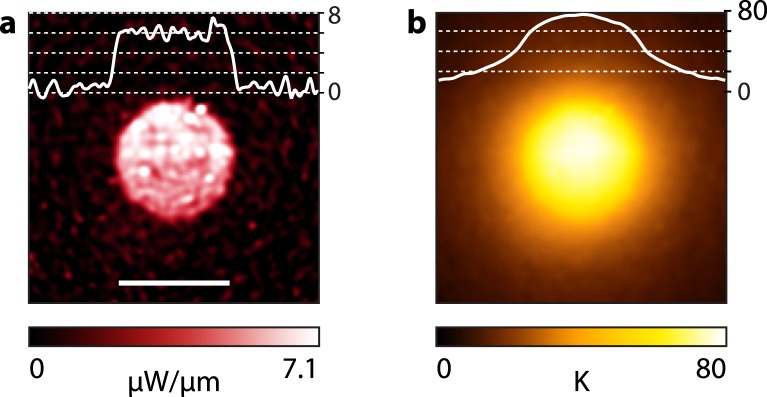
Heating of a uniform carpet of nanoparticles using a circular and uniform laser beam characterized by TIQSI. (**a**) Heat source density (scale bar 47 μm, the diameter of the laser beam). (**b**) Associated temperature distribution, featuring a non-uniform temperature distribution.

When such a gold nanoparticle substrate is illuminated using a uniform laser beam profile, the heat source density is perfectly uniform due to the uniform distribution of nanoparticles. Importantly, despite the discrete and nanometric nature of the sources of heat (the nanoparticles), the temperature profile does not consist of an assembly of nanoscale hot spots. On the contrary, the temperature distribution is smooth and continuous over the heated area, i.e., over 10 s of microns, due to collective thermal effects^27^. Thus, the heated area can be considered as a microscale hot plate delivering a uniform heat power density (HPD, power per unit area). In such a condition, even if the heat power per unit area is uniform (i.e., even if the laser intensity is uniform over a given area), the temperature is not uniform. The center of the heated area is hotter that the boundary due to thermal diffusion^27,29^, as illustrated by Fig. 2. In 2014, we introduced a technique aimed at deviating from this Gaussian-like shape to obtain almost any temperature distribution on the microscale^29^. The technique consisted in shaping the HPD by illuminating non-uniform gold nanoparticle distributions. As explained above, although this technique is effective, it does not enable a *dynamic* shaping of the temperature profile. Once lithographied, the specific nanoparticle distribution results in a fixed temperature profile at a fixed location of the sample. Here, we propose another approach to spatially shape the HPD, which consists in using a *uniform* nanoparticle distribution and a *non-uniform* laser beam. Because the HPD is now produced by a laser beam, the spatial resolution of the HPD is limited by the diffraction (typically 500 nm), while it was limited by the capabilities of e-beam lithography in our previous study (typically 100 nm). For this reason, this new approach yields a slightly poorer spatial resolution compared to the lithography approach. However, this limited spatial resolution may not be a problem for many applications and this light-shaping approach enables one to dynamically position and shape microscale temperature profiles. This is a requisite for several applications, e.g., in microscale thermophoresis of colloids or cell biology, where the location and motion of colloids or cells on the sample cannot be predicted.

For a heat source located at the interface between two semi-infinite media separated by a planar interface, the temperature everywhere in the universe reads

1 $$T({\bf{r}})=[G\otimes p]\,({\bf{r}})$$

where $G({\bf{r}})=1/(4\pi \bar{\kappa }|{\bf{r}}|)$ is the thermal Green’s function and *p*(**r**) the HPD (power per unit area at the planar interface) with $\bar{\kappa }=({\kappa }_{1}+{\kappa }_{2})/2$ is the average of the two thermal conductivities of the two semi-infinite media. This Green’s function can be used when the liquid layer is much thicker than the size of the laser beam impinging on the nanoparticles, so that the liquid layer thickness can be considered as infinite. If the water layer is reduced, then the Green’s function has to be modified accordingly. It cannot be considered anymore as scaling as 1/*r* in any direction. One has to consider the three-layer Green’s function for accurate computation^26,31^. This convolution between *G* and *p* can be numerically inverted to obtain the function *p* corresponding to a desired *T*_target_ distribution throughout the interface^29^:

2 $$p({\bf{r}})={ {\mathcal F} }^{-1}[ {\mathcal F} ({T}_{{\rm{target}}}({\bf{r}}))/ {\mathcal F} (G({\bf{r}}))]$$

where ${\mathcal F}$ represents the Fourier transform. As explained above, the approach we introduce consists in reproducing this HPD using a spatially contrasted laser beam on a uniform absorbing layer, composed here of gold nanoparticles. Hence, the laser beam intensity profile *I*_laser_ (power per unit area of the laser beam, at the interface) has to reproduce the HPD *p* to within a scalar multiplication factor. This factor equals the absorbance *A* of the sample:

3 $${I}_{{\rm{laser}}}({\bf{r}})=p({\bf{r}})/A$$

The profile of laser beam can be adjusted using different approaches. We chose to use a spatial light modulator (SLM) to achieve this task. The SLM we used consisted of a 2D-matrix of liquid crystals applying a phase shift to a laser beam in reflection. When placed within the optical path of a light beam entering the objective lens of a microscope, it can be used to modify the laser intensity by only modifying its phase in the Fourier plane, i.e., at the entrance pupil of the objective lens. This is why the SLM is optically conjugated with the objective lens location in Fig. 1. To compute the phase profile Φ(**r**) to be applied in the Fourier space to generate a given light intensity profile *I*_laser_(**r**) in the sample plane, we used the Gerchberg-Saxton phase-retrieval algorithm (GSA, see further on for a more detailed discussion)^32^. This iterative algorithm enables the determination of a phase profile Φ(**r**) such that

4 $${I}_{{\rm{laser}}}({\bf{r}})= {\mathcal F} [{I}_{0}({\bf{r}})\,\exp (i{\rm{\Phi }}({\bf{r}}))]$$

where *I*_0_(**r**) is the light intensity profile impinging on the SLM.

**Figure 3:**
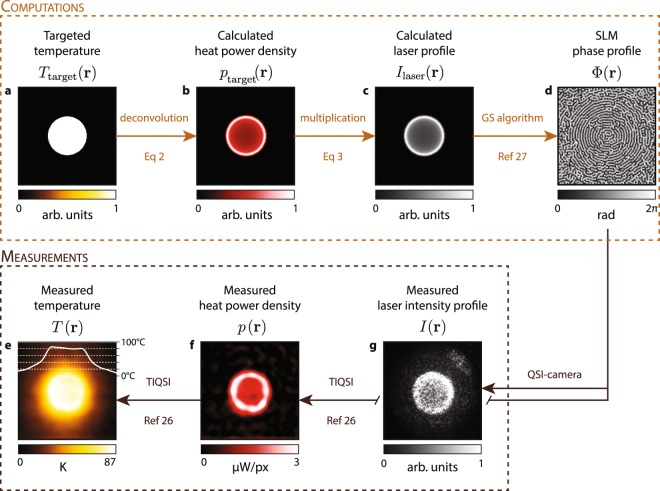
Experimental procedure of the TS-SLM technique. (**a**) Choice of a targeted microscale temperature profile. (**b**) Numerical calculation of the required heat power density to achieve this temperature profile. (**c**) Associated laser beam profile to achieve this heat power density. (**d**) Phase of the light beam at the entrance of the objective lens to achieve the laser beam profile (**c**) at the sample plane. (**e**,**f**) Experimental measurements of the temperature and heat power density using TIQSI. (**g**) Image of the light scattering by the gold nanoparticle layer to render the profile of the light beam at the sample plane. Images (**e**–**g**) have to be compared with their analog images (**a**–**c**).

Figure 3 summarizes the different steps (algorithms and experiments) to achieve temperature shaping and control using spatial light modulation (TS-SLM). The first step consists in choosing a 2-dimensional temperature profile over an area of interest. Figure 3a considers the particular example of a uniform temperature increase over a circular area. Note that some singular temperature profiles cannot be created. For instance, specifying negative temperature variations won’t obviously be possible. This would generate a calculated HPD with negative values. Also, too steep gradients may also generate negative HPD in some pixels. However, the specification of uniform temperature profiles, no matter the complexity of the shape, necessarily gives rise to valid HPD. The HPD (Fig. 3b) has to be calculated by deconvolution (see Eq. (2)). The core of the technique consists in generating this HPD by illuminating a uniform absorbing layer (here composed of gold nanoparticles) by a light pattern (Fig. 3c) that reproduces the HPD profile. The light pattern irradiance can be determined from the HPD provided the absorption of the sample is known (see Eq. (3)), which is usually not the case. This quantity is difficult to measure. One usually rather measures the extinction, which arises not only from absorption but also from scattering. But determining *A* is not a requisite in our approach. Besides, we did not do it in our study. By arbitrarily setting the laser power, the temperature distribution will eventually feature the appropriate profile, up to a constant factor, and the amplitude of the temperature increase could be adjusted a posteriori by playing with the laser power. The implementation of the temperature microscopy technique is then mandatory to monitor the overall temperature increase and adjust the laser power accordingly to achieve the appropriate temperature increase. To obtain the targeted light pattern, one option could be to use an array of micromirrors (digital light processor, DLP) but we preferred to opt for a spatial light modulator in this study. It more elegantly redistributes the light intensity in the Fourier space instead of switching pixels on and off and loosing a lot of laser intensity. A DLP would do the job, provided a laser of a few watts is used. The phase profile to be applied to the SLM to generate the appropriate light pattern was calculated using the Gerchberg–Saxton algorithm (GSA)^32^. Because no simple inversion procedure exists, GSA is based on an iterative process. The code is simple (no more than 10-line long, see Suppl. Info.), but some refinement is often implemented, in particular to remove the occurrence of speckle, inherent to this approach (see the speckle in Fig. 3g)^33,34^. But in our case, thermal diffusion smoothes any small non-uniformities of the heat generation on the microscale, so that any procedure to damp the speckle is not mandatory. It is, however, mandatory to remove the zero-order spot, that would create a heating spot in the middle of the image. For this purpose, a linear phase gradient is applied to the phase image calculated by the GSA in order to laterally shift the light pattern on the sample plane away from the zero-order of diffraction. Then, a diaphragm (D) is positioned at an intermediary image plane (see Fig. 1a) to remove this light spot and only keep the light pattern of interest.

Once the appropriate light pattern is projected onto the sample, the resulting temperature field has to be controlled. For this purpose, many temperature microscopies have been developed, most of them based on optical measurements, usually involving fluorescence (see Chap. 4 of ref.^1^). In this study, we used QSI as it is label-free: it does not involve the addition of any molecular temperature probe since the measurement is based on temperature-induced variations of the refractive index of the liquid layer^30^. Moreover, this technique enables not only the measurement of the temperature, but also of the HPD, quantitatively. Such measurements are presented in Fig. 3e,f, and they are in agreement with the targeted temperature and HPD (see Fig. 3a,b). QSI also enables the acquisition of intensity images (not only phase), as displayed in Fig. 3g. In that case, the optical filter was changed in front of the camera to remove the light from the Köhler illumination and let the laser pass.

Gold nanoparticles have been our preferred choice for this study. Although any two-dimensional, uniform absorbing layer should be effective as well, our choice was driven by the multiple benefits provided by gold nanoparticles: First, when using sufficiently small nanoparticles (typically below 40 nm), it is possible to make absorption the only interaction process existing between light and nanoparticles: (i) Scattering can be made negligible because it scales as the volume squared, while the absorption scales with the volume of the nanoparticle. Then, no reflection of the laser beam occurs like what would happen with a metal layer for instance, acting as a mirror. Thus, the transduction of the light energy can be optimized, with no light energy lost in unwanted side-effects. In our case, gold nanorods 46 × 20 nm in size feature an absorption/scattering ratio of 7 at resonance.

Second, unlike dyes, gold nanoparticles do not photobleach, do not oxidize, and get damaged/reshaped only at temperatures higher that 150 °C. This makes gold nanoparticles samples particularly stable and robust. Third, although gold is an expensive metal, several inexpensive bottom-up fabrication approaches exist to yield uniform distributions of nanoparticles over macroscopic areas. Fourth, gold nanoparticles can feature resonant absorption in the infrared, if they deviate from a spherical geometry, like in this study. This offers the possibility to build a sample that is absorbant in the infrared, but which remains quite transparent in the visible range. This is highly desirable to ease any fluorescence imaging for exemple.

The only limitation of using gold nanoparticles is that they can reshape when typically exceeding 150 °C to 200 °C (under cw illumination) depending on the heating duration^35^. Reshaping induces a variation of their absorption efficiency, which would compromise the required uniformity of the sample. To avoid this problem in high-temperature applications, the gold nanoparticle layer may be embedded in a silica matrix^36^.

**Figure 4:**
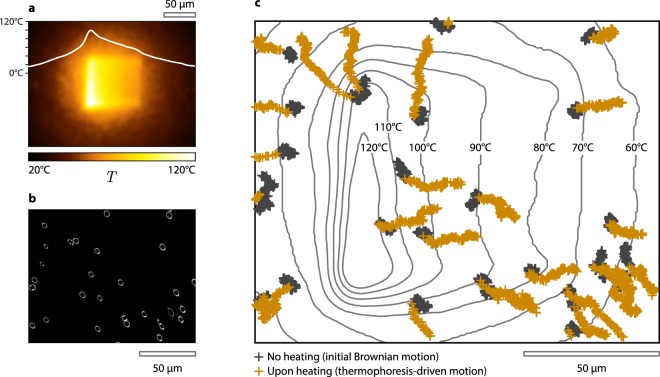
Thermophoresis-driven motion of 28 beads. (**a**) Linear temperature gradient produced by TS-SLM. (**b**) Dark-field image of beads dispersed in the liquid layer. (**c**) Trajectory of the beads with no heating during 14 seconds (dark crosses) exhibiting a Brownian motion, and upon heating (brown crosses) undergoing a linear motion following the temperature gradient.

In the previous part, we chose to illustrate the principle of the TS-SLM approach on a uniform temperature distribution because it is particularly suited for experiments in chemistry^20^ and cell biology^4^, where the setting of a precise temperature over the observation area may be mandatory. But another identified relevant temperature profile for other applications consists of *linear temperature gradients*, e.g., for the study of microscale thermophoresis in liquids^16,28,37^. This is what we wish to evidence in Fig. 4. In this experiment, silica beads, 1.24 μm in diameter, were dispersed in milli-Q water, with a fluid thickness of 34 μm. Reducing the thickness of the fluid enables the damping of fluid convection, that would cancel the thermophoretic motion of the beads. Figure 4a displays a linear temperature gradient of 0.36 K/μm applied over a rectangular area by TS-SLM. In this experiment, a super-heated state of water (water above 100 °C without boiling) was achieved^15^. Figure 4b is a dark-field image of the beads dispersed in water. When no heating is performed, these beads are simply undergoing Brownian motion at ambient temperature (21 °C). When the linear temperature gradient is applied, a strong thermophoretic motion is observed, moving the beads from hot to cold. Video V1, provided in Suppl. Info., shows such a process. During the first 14 s, no heating was applied. Then, heating was applied during 14 s, and the beads exhibited a drift. At this scale, the temperature distribution observed in Fig. 4a establishes on the millisecond scale, and can be considered as instantaneous, compared to the time scale of the physical process under consideration. Figure 4c represents the motion of the beads of Fig. 4b and in the video. Dark crosses correspond to the no-heating phase, lasting 14 s. No drift in specific direction is observed, only Brownian motion. The brown crosses correspond to the first 14 s of heating, where clear directed motions are observed, along the temperature gradient. The four beads in the centre of the image, where the temperature gradient is linear, enable the quantification of the thermophoretic properties of the beads. In this temperature range (80–120 °C), the measured thermophoretic mobility *D*_*T*_ is 3.5 ± 0.6 μm^2^/(K · s). Refined measurements could be performed with statistics on a larger number of beads, but this will be the purpose of a future work dedicated to microscale thermophoresis in liquids. The idea here was to illustrate the applicability and the interest of TS-SLM for an original application.

## Conclusions

In summary, we introduced an optical microscopy technique suited to generate microscale temperature patterns with arbitrary shapes, in particular uniform distributions and linear temperature gradients, the most useful cases for envisioned applications. This technique involves a spatial light modulator (to generate the temperature profile) and a wavefront sensing camera (to measure the temperature profile) or any other temperature mapping technique. This approach enables a dynamic control of the temperature profile, even down to the millisecond scale. This experimental scheme can be simply implemented on any regular microscope. Applications are envisioned when an accurate temperature setting over the observation area of interest in mandatory, for instance in microscale thermal-assisted chemistry, fluid dynamics, cell biology and more generally in most applications of thermoplasmonics.

## Methods

### Gold nanoparticle sample fabrication

The gold nanorods synthesis is adapted from the protocols developed by Nikoobakht *et al*.^38^ and Liu *et al*.^39^, and detailed in ref.^4^. The as-prepared gold nanorods were functionalized with PVP. Before the deposition of the PVP-functionalized gold nanorods onto the surface of the coverslip, the latter has been recovered by 3 layers of polyelectrolytes as described in the work of Vial *et al*.^40^. The functionalized coverslips were immersed in a solution of PVP-gold nanorods for 3 hours. The deposition was promoted via electrostatic interactions between the positively charged coverslip and the negatively charged gold nanorods.

## Supplementary information

Matlab programs

Video
