# Supplementary material for: Microscale Temperature Shaping Using Spatial Light Modulation on Gold Nanoparticles
Source: Sci Rep. 2019 Mar 15;9:4644. doi: 10.1038/s41598-019-40382-3 (PMC6420633; doi:10.1038/s41598-019-40382-3)
Supplement: Supplementary file 1 — Matlab programs [file 41598_2019_40382_MOESM1_ESM.docx]

**Microscale Temperature Shaping**

**Using Spatial Light Modulation on Gold Nanoparticles**

Ljiljana Durdevic,^1^ Hadrien M. L. Robert, ^1^ Benoit Wattellier, ^2^ Serge Monneret, ^1^ Guillaume Baffou^1^

^1^*Institut Fresnel, CNRS, Aix Marseille Univ, Centrale Marseille, Marseille, France*

^2^*PHASICS S.A., Parc technologique de Saint Aubin, Route de l'Orme des Merisiers, 91190 Saint Aubin, France*

**SUPPLEMENTARY INFORMATION**

Matlab code for generating an SLM interferogram

associated to a targeted temperature profile.

main.m

%% Typical main program to pilot an SLM and generate arbitrary temperature

%% profiles via a microscope.

% Guillaume Baffou & Ljiljana Durdevic

% CNRS - Institut Fresnel, Marseille (France)

% 26 August 2018

%%%%%%%%%%%%%%%%%%%%%%%%%%%%%%%%%%%%%%%%%%%%%%%%

% 1a- Fabrication of the profile of the laser beam meant to create

% a predefined temperature distribution, here a uniform disc.

%Qmap=uniformSquare(100,100,20,1);

% 1b- an alternative approach consists in using a jpg or bmp B&W image.

% What is black is what is supposed to be hot (see the example file)

Qmap=importImage('S.jpeg',1);

% 2- Computation of the phase profile to be sent to the SLM to create the

% above-calculated light profile at the sample plane.

hologram=GerchbergSaxton(Qmap);

GerchbergSaxton.m

%% Gerchberg Saxton algorithm

% Guillaume Baffou & Ljiljana Durdevic

% 28 March 2018 - Institut Fresnel, CNRS, Marseille (France)

% Nanoscale 6, 8984 - 8989 (2014)

function hologram=GerchbergSaxton(Target)

[Ny, Nx]=size(Target);

%Source=Target*0+1; % Profile of the laser impinging on the SLM. 1 for uniform.

Ny2=Ny/2;

Nx2=Nx/2;

r2=(min([Nx/2,Ny/2]))^2;

Source=Target*0;

for x=-Nx2+1/2:1:Nx2-1/2

for y=-Ny2+1/2:1:Ny2-1/2

if x*x+y*y<r2;

ix=x+Nx2+1/2;

iy=y+Ny2+1/2;

Source(iy,ix)=1;

end

end

end

A=fftshift(ifft2(fftshift(Target))); %Fourier

for i=1:10

B = abs(Source) .* exp(1i*angle(A)); %Fourier

C = fftshift(fft2(fftshift(B))); %Real space

D = abs(Target) .* exp(1i*angle(C));

A = fftshift(ifft2(fftshift(D)));

%imagesc(angle(C)) %Present current pattern

%title(sprintf('%d',i));

%pause(0.5)

end

hologram=A;

%%

B = abs(Source) .* exp(1i*angle(hologram)); %Fourier

C = fftshift(fft2(fftshift(B))); %Real space

figure, imagesc(abs(C))

title('Intensity profile in the imaging plane');

axis image;

figure, imagesc(angle(hologram))

title('Phase image to be sent to the SLM');

colormap(hsv)

colorbar

axis image;

fprintf('Done\n')

importImage.m

%% Calculation of the HSD map from an imported B&W image.

%% Meant to create uniform temperature profiles.

% Guillaume Baffou 28/03/2018 -Institut Fresnel, CNRS, Marseille (France)

function Qmap=importImage(fileName,smooth)

% fileName: a string that corresponds to the file Name of the image to be

% imported. It can be in B&W, rgb, jpg, bmp, png, ...

% smooth : Gaussian smoothing factor. By default, 1.

if nargin~=3

smooth=1;

end

%% importation of the image

IM=double(imread(fileName));

dim=length(size(IM));

maxVal=max(IM(:));

if dim==3

IM=(IM(:,:,1)+IM(:,:,2)+IM(:,:,3))/3;

end

% processing of the image so that the pixel values are 0 or 1.

IM=IM-maxVal;

IM=-IM;

IM=round(IM/maxVal);

figure,imagesc(IM)

title('Target uniform temperature profile')

axis image;

%% processing of the laser beam profile

[Ny, Nx]=size(IM);

n=0;

Nx2=Nx/2;

Ny2=Ny/2;

for x=-Nx2+1/2:1:Nx2-1/2

for y=-Ny2+1/2:1:Ny2-1/2

ix=x+Nx2+1/2;

iy=y+Ny2+1/2;

if IM(iy,ix)~=0

n=n+1;

Tline(n)=IM(iy,ix);

rx(n)=x;

ry(n)=y;

end

end

end

AA=zeros(n,n);

for n1=1:length(rx)

for n2=1:length(rx)

AA(n1,n2)=1/sqrt((rx(n1)-rx(n2))^2+(ry(n1)-ry(n2))^2);

end

end

for n=1:length(rx)

AA(n,n)=2;

end

Qline=AA\Tline';

Qmap=zeros(size(IM));

for n=1:length(rx)

Qmap(ry(n)+Ny2+1/2,rx(n)+Nx2+1/2)=Qline(n);

end

Qmap=imgaussfilt(Qmap,smooth);

figure,imagesc(Qmap)

axis image;

title('Laser beam profile')

dlmwrite('Qmap.txt',Qmap,' ')

%% calculation of the resulting T map

% (optional part, just to have an idea

% of the final 2D temperature profile)

NxBig=2*Nx;

NyBig=2*Ny;

QmapBig=zeros(NyBig,NxBig);

QmapBig(floor(Ny2+1:Ny2+Ny),floor(Nx2+1:Nx2+Nx))=Qmap;

rBigx=ones(NyBig,1)*(-Nx+1/2:1:Nx-1/2);

rBigy=(-Ny+1/2:1:Ny-1/2)'*ones(1,NxBig);

Green=1./sqrt(rBigx.^2+rBigy.^2);

TmapBig=conv2(Green,QmapBig);

TmapAll=TmapBig(Ny+1:Ny+NyBig,Nx+1:Nx+NxBig);

figure,imagesc(TmapAll)

axis image;

title('Temperature profile')

dlmwrite('Tmap.txt',TmapAll,' ')

dlmwrite('Tprofile.txt',TmapAll(Ny,:),'\n')

dlmwrite('Qprofile.txt',QmapBig(Ny,:),'\n')

dlmwrite('r.txt',(-Ny+1:Ny-1)')

uniformDisc.m

%% Calculation of the HSD map from a predefined T map

% Guillaume Baffou 28/03/2018 -Institut Fresnel, CNRS, Marseille (France)

function Qmap=uniformDisc(Nx,Ny,radius,smooth)

% Nx, Ny: Size of the image, in px.

% Must be even numbers.

% radius: radius of the uniform-temperature disc, in px.

% smooth: Gaussian smoothing factor. By default, 1.

if nargin~=3

smooth=1;

end

Tmap=zeros(Ny,Nx);

Nx2=Nx/2;

Ny2=Ny/2;

%% definition of the temperature field over an area of interest

% in this example : uniform temperature = 1 for r < radius.

n=0;

for x=-Nx2+1/2:1:Nx2-1/2

for y=-Ny2+1/2:1:Ny2-1/2

if x*x+y*y<radius^2;

n=n+1;

ix=x+Nx2+1/2;

iy=y+Ny2+1/2;

Tmap(iy,ix)=1;

Tline(n)=1;

rx(n)=x;

ry(n)=y;

end

end

end

% determination of the laser beam profile

AA=zeros(n,n);

for n1=1:length(rx)

for n2=1:length(rx)

AA(n1,n2)=1/sqrt((rx(n1)-rx(n2))^2+(ry(n1)-ry(n2))^2);

end

end

for n=1:length(rx)

AA(n,n)=2;

end

Qline=AA\Tline';

Qmap=zeros(size(Tmap));

for n=1:length(rx)

Qmap(ry(n)+Ny2+1/2,rx(n)+Nx2+1/2)=Qline(n);

end

Qmap=imgaussfilt(Qmap,smooth);

imagesc(Qmap)

axis image;

dlmwrite('Qmap.txt',Qmap,' ')

%% calculation of the resulting T map

% (optional part, just to have an idea

% of the actual 2D temperature profile)

NxBig=2*Nx;

NyBig=2*Ny;

QmapBig=zeros(NyBig,NxBig);

QmapBig(Ny2+1:Ny2+Ny,Nx2+1:Nx2+Nx)=Qmap;

rBigx=ones(NyBig,1)*(-Nx+1/2:1:Nx-1/2);

rBigy=(-Ny+1/2:1:Ny-1/2)'*ones(1,NxBig);

Green=1./sqrt(rBigx.^2+rBigy.^2);

TmapBig=conv2(Green,QmapBig);

TmapAll=TmapBig(Ny+1:Ny+NyBig,Nx+1:Nx+NxBig);

figure,imagesc(TmapAll)

axis image;

figure,plot(TmapAll(Ny,:))

figure,plot(QmapBig(Ny,:))

dlmwrite('Tmap.txt',TmapAll,' ')

dlmwrite('Tprofile.txt',TmapAll(Ny,:),'\n')

dlmwrite('Qprofile.txt',QmapBig(Ny,:),'\n')

dlmwrite('r.txt',(-Ny+1:Ny-1)')

uniformSquare.m

%% Calculation of the HSD map from a predefined T map

% Guillaume Baffou 28/03/2018 -Institut Fresnel, CNRS, Marseille (France)

function Qmap=uniformSquare(Nx,Ny,sqSize,smooth)

% Nx, Ny: Size of the image, in px.

% Must be even numbers.

% sqSize: size of the uniform-temperature square, in px.

% smooth: Gaussian smoothing factor. By default, 1.

if nargin~=3

smooth=1;

end

Tmap=zeros(Ny,Nx);

Nx2=Nx/2;

Ny2=Ny/2;

%% definition of the temperature field

%% over an area of interest

% in this example : uniform temperature = 1 over a square.

n=0;

for x=-Nx2+1/2:1:Nx2-1/2

for y=-Ny2+1/2:1:Ny2-1/2

if abs(x)<sqSize/2 && abs(y)<sqSize/2;

n=n+1;

ix=x+Nx2+1/2;

iy=y+Ny2+1/2;

Tmap(iy,ix)=1;

Tline(n)=1;

rx(n)=x;

ry(n)=y;

end

end

end

% determination of the laser beam profile

AA=zeros(n,n);

for n1=1:length(rx)

for n2=1:length(rx)

AA(n1,n2)=1/sqrt((rx(n1)-rx(n2))^2+(ry(n1)-ry(n2))^2);

end

end

for n=1:length(rx)

AA(n,n)=2;

end

Qline=AA\Tline';

Qmap=zeros(size(Tmap));

for n=1:length(rx)

Qmap(ry(n)+Ny2+1/2,rx(n)+Nx2+1/2)=Qline(n);

end

Qmap=imgaussfilt(Qmap,smooth);

imagesc(Qmap)

axis image;

dlmwrite('Qmap.txt',Qmap,' ')

%% calculation of the resulting T map

% (optional part, just to have an idea

% of the actual 2D temperature profile)

NxBig=2*Nx;

NyBig=2*Ny;

QmapBig=zeros(NyBig,NxBig);

QmapBig(Ny2+1:Ny2+Ny,Nx2+1:Nx2+Nx)=Qmap;

rBigx=ones(NyBig,1)*(-Nx+1/2:1:Nx-1/2);

rBigy=(-Ny+1/2:1:Ny-1/2)'*ones(1,NxBig);

Green=1./sqrt(rBigx.^2+rBigy.^2);

TmapBig=conv2(Green,QmapBig);

TmapAll=TmapBig(Ny+1:Ny+NyBig,Nx+1:Nx+NxBig);

figure,imagesc(TmapAll)

axis image;

figure,plot(TmapAll(Ny,:))

figure,plot(QmapBig(Ny,:))

dlmwrite('Tmap.txt',TmapAll,' ')

dlmwrite('Tprofile.txt',TmapAll(Ny,:),'\n')

dlmwrite('Qprofile.txt',QmapBig(Ny,:),'\n')

dlmwrite('r.txt',(-Ny+1:Ny-1)')

S.jpg

http://guillaume.baffou.com/images/S.jpg

S.bmp

http://guillaume.baffou.com/images/S.bmp
